# Development and validation of a system for the prediction of challenging behaviors of people with autism spectrum disorder based on a smart wearable shirt: A mixed-methods design

**DOI:** 10.3389/fnbeh.2022.948184

**Published:** 2022-11-29

**Authors:** Moti Zwilling, Alberto Romano, Hay Hoffman, Meir Lotan, Riki Tesler

**Affiliations:** ^1^Department of Economics and Business Administration, Ariel University, Ariel, Israel; ^2^Department of Health System Management, Ariel University, Ariel, Israel; ^3^Department of Computer Science, Ariel University, Ariel, Israel; ^4^Department of Physical Therapy, School of Health Sciences, Ariel University, Ariel, Israel

**Keywords:** autism spectrum disorder, adult, problem behavior, wearable electronic devices, accident prevention, recurrent neural network

## Abstract

**Background:**

Most people with autism spectrum disorder (ASD) present at least one form of challenging behavior (CB), causing reduced life quality, social interactions, and community-based service inclusion.

**Objectives:**

The current study had two objectives: (1) to assess the differences in physiological reaction to stressful stimuli between adults with and without high-functioning ASD; (2) to develop a system able to predict the incoming occurrence of a challenging behaviors (CBs) in real time and inform the caregiver that a CB is about to occur; (3) to evaluate the acceptability and usefulness of the developed system for users with ASD and their caregivers.

**Methods:**

Comparison between physiological parameters will be conducted by enrolling two groups of 20 participants with and without ASD monitored while watching a relaxing and disturbing video. To understand the variations of the parameters that occur before the CB takes place, 10 participants with ASD who have aggressive or disruptive CBs will be monitored for 7 days. Then, an ML algorithm capable of predicting immediate CB occurrence based on physiological parameter variations is about to be developed. After developing the application-based algorithm, an efficient proof of concept (POC) will be carried out on one participant with ASD and CB. A focus group, including health professionals, will test the POC to identify the strengths and weaknesses of the developed system.

**Results:**

Higher stress level is anticipated in the group of people with ASD looking at the disturbing video than in the typically developed peers. From the obtained data, the developed algorithm is used to predict CBs that are about to occur in the upcoming 1 min. A high level of satisfaction with the proposed technology and useful consideration for further developments are expected to emerge from the focus group.

**Clinical trial registration:**

[https://clinicaltrials.gov/], identifier [NCT05340608].

## Introduction

Autism spectrum disorder (ASD) refers to a heterogeneous neurodevelopmental condition with symptoms that range from mild to severe. ASD is generally detected in childhood and is lifelong. It affects between 1 and 2% of the population (Baron-Cohen et al., 2009) and is characterized by social communication deficits, repetitive and unusual sensory-motor behaviors, and restricted and specific interests (American Psychiatric Association, 2013). The literature indicates that about 55% of the people diagnosed with ASD also exhibit intellectual disabilities (Knapp et al., 2009), and about 25% are non or minimally verbal (Arnold and Reed, 2016).

Challenging behaviors (CBs) (Murphy et al., 2005; Prizant and Wetherby, 2005; Reese et al., 2005; Baker et al., 2008; Chiang, 2008; O’Donnell et al., 2012) refer to a broad range of unusual behaviors expressed by individuals with ASD. Such behaviors might include aggression, destructiveness, self-injurious, and a range of other behaviors, such as unacceptable social and sexual conduct (Emerson, 2001; Holden and Gitlesen, 2006). Most studies reported high rates of CBs among individuals with ASD, with a prevalence of up to 94% presenting at least one type of challenging behavior (CB) (Matson et al., 2008; Jang et al., 2011). Other studies have reported on the appearance of CBs among 82% of participants, with 32.5% involving aggressive behavior toward themselves or others (Woodbury-Smith et al., 2006; Murphy et al., 2009). CBs may significantly impair the physical and mental health and the quality of life of the persons presenting such behaviors, those who care for them, those who manage them (therapists; teachers), and even their neighbors (Nissen and Haveman, 1997; Blacher and McIntyre, 2006; Mukaddes and Topcu, 2006; Felce et al., 2011).

Applying therapeutic strategies in such cases is strongly warranted to prevent the CBs from becoming a part of an individual’s behavioral repertoire. In the absence of therapeutic strategies, such behaviors are unlikely to decrease and will typically remain or worsen without intervention (Berg et al., 2000). An effective intervention to reduce the outburst of CBs and one that could possibly lessen the severity of a CB could enhance the involvement of the person with ASD within society and reduce the financial and emotional burden of the family and caregivers while simultaneously decreasing the need for medications (Rose et al., 2004).

Several forms of intervention have been proposed for reducing CB in people with ASD, including medications (Malone et al., 2005; Blankenship et al., 2010; McPheeters et al., 2011; Sawyer et al., 2014), behavioral interventions (Scotti et al., 1996; Didden et al., 2006; Machalicek et al., 2007, 2016; Lydon et al., 2013; Fettig and Barton, 2014; Erturk et al., 2018; MacNaul and Neely, 2018; Weston et al., 2018; Inoue, 2019), cognitive/emotion-oriented interventions (Neal and Barton Wright, 2003; Zetteler, 2008; O’Neil et al., 2011; Cotelli et al., 2012; Subramaniam and Woods, 2012; Doyle et al., 2013), sensory stimulation/integration interventions (Lang et al., 2012; Barton et al., 2015; Case-Smith et al., 2015; Leong et al., 2015; Wan Yunus et al., 2015; Watling and Hauer, 2015), music therapy (Gold et al., 2006; Stephenson, 2006; Simpson and Keen, 2011; James et al., 2015; Fakhoury et al., 2017), psychosocial interventions (Seida et al., 2009; Reichow et al., 2013; Vanderkerken et al., 2013; Bishop-Fitzpatrick et al., 2014; Lim, 2019), communication training (Mirenda, 1997; Goldstein, 2002; Lequia et al., 2012; Walker and Snell, 2013; Gerow et al., 2018; Gregori et al., 2020), physical exercises (Eggermont and Scherder, 2006; Ogg-Groenendaal et al., 2014; Sorensen and Zarrett, 2014; Forbes et al., 2015; Bremer et al., 2016), and others (McDonnell et al., 2008; Tanner et al., 2015; Lindgren et al., 2016; Ferguson et al., 2019; Walker et al., 2021; Wahman et al., 2022). Despite the wide availability of intervention forms, no consensus has been reached concerning the global efficacy of any CB treatment in treating all the CBs types. As both desirable and undesirable behaviors are learned and maintained through interaction with the social and physical environment, the behavior-environment interaction can be described as positive or negative behavior contingencies. Experts agree in affirming that a better understanding of behavior-environment relations may lead to more effective interventions (Lloyd and Kennedy, 2014). Such knowledge can be obtained by analyzing the function of the behavior. Functional behavior assessment (FBA) enables hypotheses about the relations among specific types of environmental events and behaviors. The idea behind FBA is that if these reinforcement contingencies can be identified, then interventions can be designed to decrease problem behavior and increase adaptive behavior by altering these contingencies (Cooper et al., 2020). Reinforcement contingencies maintaining CBs include positive and negative reinforcement. Positive reinforcements comprise social positive reinforcements (attention), tangible reinforcements (items or activities), and automatic positive reinforcements (engaging in the behavior itself, independently from the social environment). Negative reinforcements include social negative reinforcement (escape from socially mediated stimuli), and automatic negative reinforcement (escape from non-socially mediated stimuli such as pain) (Lloyd and Kennedy, 2014; Cooper et al., 2020). Evidence from the literature suggests that interventions based on functional assessment outcomes are more effective than those that are not function-based. Most interventions for CBs aim to prevent the occurrence of CBs themselves by guiding the person toward more adaptive behaviors while avoiding managing the consequences of CBs.

In line with the need for effective prevention strategies, the literature has been recently enriched with the proposal of using technological devices to predict CB occurrence based on the physiological parameters of the individual with ASD. The presence of atypical physiological arousal in people with ASD has been known for a long time, and the functional relation between homeostatic regulation and CB has already been hypothesized (Hutt and Hutt, 1965; Ornitz and Ritvo, 1968; Kinsbourne, 1980). More recently, atypical autonomic reactivity was reported as a common feature in people with ASD (Cohen et al., 2011; Levine et al., 2014; Klusek et al., 2015; Lydon et al., 2016). The use of physiological-biological signals such as the electrocardiogram, heart rate (HR), HR variability, respiratory rate (as well as changes in respiratory rate), and body movements are reiterated in several articles as markers for CB expressed in people with ASD (Goodwin et al., 2018, 2019; Ozdenizci et al., 2018; Taj-Eldin et al., 2018; Nuske et al., 2019).

Smart wearable shirts (SWS) are wearable medical devices that are considered to be a technological breakthrough, enabling continuous surveillance of human vital physiological signs without any disturbance to the activities of daily living. The SWS technology has been used in clinical research for the last two decades. In the previous few years, SWS have enabled the collection of varied physiological data outside the laboratory for a prolong time such as weeks at a time. The constant surveillance enabled by these devices allows for identifying physiological anomalies that deviate from the typical individual’s behaviors that can be received, analyzed, and treated (Banaee et al., 2013). Furthermore, garments, such as t-shirts, have been found to be a highly preferred device to be used by individuals with ASD (Koo et al., 2018), with a moderate to high suitability index for this population (Taj-Eldin et al., 2018). Moreover, few studies have used SWS among individuals with ASD (Taj-Eldin et al., 2018; Black et al., 2020).

The analysis of physiological data can be achieved by machine learning (ML). ML is a method that provides automated approaches for data analysis (Murphy, 2012). It utilizes machine-constructed algorithms that detect specific patterns in the data through a training process (Gulshan et al., 2016). The use of ML approaches to predict CBs occurrence has increased in recent years (Francese and Yang, 2021). Masino et al. (2019) evaluated the accuracy of support vector machine (SVM) and logistic regression (LR) classifiers in differentiating physiological states associated with stressful and non-stressful scenarios in children with ASD in a controlled laboratory setting using wearables data. The authors reported on higher accuracy of the SVM classifier and suggested that ML models combined with wearables data may support real-time intervention in the population with ASD. Imbiriba et al. (2020) reported similar results when using an SVM combined with a principal component analysis (PCA) model to predict aggression in youth with ASD. The authors stated the adequacy of the model to predict aggression 3 min before their appearance. Moreover, higher prediction performance was reported for the SVM + PCA model than for the LR model. Consistently, Cantin-Garside et al. (2021) reported higher accuracy of SVM and k-nearest neighbor (kNN) algorithm in classifying self-injurious behavior in children with ASD compared to other methods [discriminant analysis (DA), decision trees (DT), Naïve Bayes (NB), and neural networks (NN)]. Furthermore, Zheng et al. (2021) proposed a multimodal data analysis to predict precursors of CBs of children with ASD through various ML algorithms. Their multimodal data capture platform is composed of wearable bio (peripheral physiological signals) and gesture (acceleration signals) sensors combined with Kinect cameras (facial expressions and head rotations). The study results pointed on a higher prediction accuracy for random forest (RF) and NN algorithms compared to SVM, DA, kNN, DT, and NB algorithms when looking for precursors of CBs. Although referred to the pediatric population only, these preliminary insights support using ML algorithms and wearable devices to predict CBs in people with ASD.

The current protocol consists of three phases, each with a specific goal. The first aim is to assess the differences in the measured physiological reaction between adults with high-functioning ASD and their typically developed peers utilizing SWS. The second goal is to create an *ad hoc* ML algorithm that will be utilized for real-time CB prediction and combined with a smartphone application that sends an alert when the CB is likely to occur. Finally, we aimed to test the developed system among people with ASD and assess its acceptability and usefulness for users with ASD and their caregivers.

## Materials and methods

### Study design

An observational study design will be implemented in the first two phases of the current research. In phase one, participants’ (with and without ASD) physiological reactions to two visual stimuli (pleasant vs. disturbing) will be collected and analyzed. The physiological characteristics of the CBs presented by people with ASD will be collected in phase two, coupled with behavioral diaries filled out by the care providers. Finally, a single case study with a mixed-method design will be implemented in phase three, where the system validity proof of concept (POC) will be performed.

### Ethics and safety issues

The research proposal was approved by the Ariel University Institutional Review Board (AU-HEA-ML-20201203), Asaf Harofe Institutional Review Board (0136-21-ASF), and the Israeli Ministry of Health (MOH_2022-01-25_010570). The implementation of the protocol was also approved by the head scientist from the Israeli Ministry of Social Affairs and Welfare. The trial protocol was registered in the World Health Organization Trial Registry (ClinicalTrials.gov ID: NCT05340608). Written consent was also given by the head of the residential centers hosting the second section of the proposed study. The study will be carried out following the Declaration of Helsinki principles. Written informed consent will be collected from all participants or their legal guardians at the recruitment stage. The SWSs planned to be used are non-invasive medical devices with sensors that collect physical signals from the participants. However, if a participant refuses to wear the SWS, he or she may withdraw from the study at any time without any repercussions.

### Participants

According to the sample size calculation performed, a group of 20 subjects diagnosed with high-functioning ASD aged between 20 and 40 years residing at home [observation group (OG)], along with an age- and sex-matched control group (CG) of 20 typically developed peers, will be enrolled in the first protocol phase. In the second phase, 10 people with ASD presenting with intensive aggressive or disruptive CBs aged 20–40 years and their caregivers will be recruited. Finally, one participant with ASD aged 20–40 years exhibiting aggressive or disruptive CBs will participate in the third phase of the research as POC.

### Outcome measures

#### Smart wearable shirt

The Hexoskin SWS (Hexoskin Inc., Montreal, QC, Canada) is a wearable device with several sensors to measure physiological signals. Its producer declares the SWS as a non-invasive SWS with textile-embedded sensors that allow the collection of multiple parameters. A detailed description of sensors equipped in the Hexoskin SWS is available on the producer’s website.^1^ The Hexoskin SWS will be used in all three phases of the research.

#### Behavioral diary

The care providers of participants enrolled in phase two will be asked to fill out a daily behavioral diary reporting the arousal level of each participant. Three arousal levels will be collected: quiet, agitated, and CB. The “quiet” state refers to a period in which the subject is relaxed or calmly going about his or her daily routine (e.g., resting on the sofa). Being “agitated” describes a behavioral activation state higher than “quiet”. It can correspond to situations in which a physiological reaction is observed, such as redness, sweating, and increased respiratory rate, among others. It can occur in cases of euphoria (e.g., the subject is watching a show that he or she extremely enjoys); intense activity (e.g., doing a sport activity); or anger (e.g., the subject has been told that he or she cannot do an activity that he or she has requested and therefore vigorously protests), but cannot be defined as CB. “CB” state describes extreme agitation and an intense physiological response (redness, sweating, or increased respiratory rate). It can be accompanied by fierce anger (with or without aggressive or disruptive behaviors), strong states of anxiety, or a need to move intensely. In general, “CB” should correspond to reactions identified as exaggerated, excessive for the situation, or inadequate relative to the social context. Aggression behaviors will include self or other-directed physical or verbal aggression. In some cases, such behaviors may be uncontrolled by the participants. For each arousal level reported, caregivers will be asked to report on the following items: the beginning and ending time and date, arousal level, and operational definition of the accompanying behavior and activities. The behavioral diary will be collected within the second phase.

#### Quebec user evaluation of satisfaction with assistive technology

The Quebec User Evaluation of Satisfaction with Assistive Technology second edition (QUEST 2.0) (Demers et al., 2000) is a 12-item questionnaire designed to assess users’ satisfaction with a wide range of assistive technology (Scherer, 2005). The 12 items are grouped into two areas representing user satisfaction with the assistive technologies related to the assistive device (eight items) and provided service (four items). A five-point Likert scale is given to each item, ranging from one (“not satisfied at all”) to five (“very satisfied”). Strong psychometric proprieties have been published for the QUEST 2.0 (Demers et al., 2002). The QUEST 2.0 will be administered in phase three of the current protocol by participants’ caregivers.

#### Focus group

A focus group is a qualitative data collection method often used in health research. The technique is used to produce a controlled discussion on specific issues within a group of people who share different experiences or relations with the focused topics (Kitzinger, 1994; Flores and Alonso, 1995). Under the focus group method, the group discussion is recorded, transcribed, and analyzed. In addition, a search for themes relevant to the investigated topic and the group agreement assessment is performed (Breen, 2006). Research questions that will be raised during the focus group include:

• Did wearing the SWS upset the participants?
• Was the system able to detect all relevant CBs?
• Was the system’s operational speed sufficient to allow the in-time application of appropriate prevention strategies?
• Has the use of the system reduced the amount of CBs?
• What improvements can be applied to the system to increase its effectiveness?

### Procedure

**FIGURE 1:**
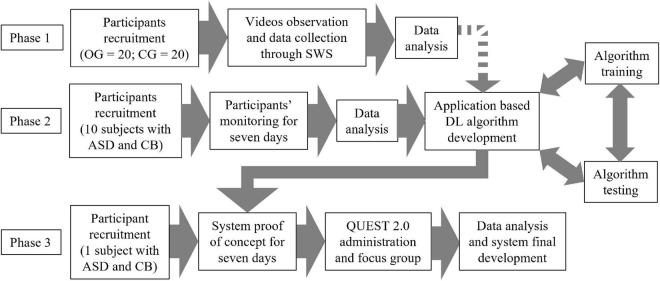
Protocol flowchart. The dotted arrow indicates that the integration of the data obtained from phase one will only occur if they bring added value to the development of the algorithm. OG, observation group; CG, control group; SWS, smart wearable shirt; ASD, autism spectrum disorder; DL, deep learning; CB, challenging behavior; QUEST 2.0, Quebec User Evaluation of Satisfaction with Assistive Technology 2nd edition.

The research procedure is outlined in Figure 1. The protocol’s expected start date is June 2022.

#### Phase one – Comparison of physiological outcomes between people with and without autism spectrum disorder

For the first phase, the physiological parameters of the people in the OG and CG will be acquired and recorded using the Hexoskin SWS while participants watch two different 5 min videos. One video will show relaxing images while emitting relaxing music (relaxing video). The second video will present human facial deformities accompanied by anxious music (disturbing video). Both videos will be presented to the participant when in a seated position. Before starting the relaxing video, the participant will be invited to relax and lean back onto the chair’s backrest. The participant can close his eyes or keep them open at his or her discretion to promote relaxation. To watch the disturbing video, participants will be asked not to lean against the chair backrest and keep their eyes open for the duration of the video. The video viewed by each participant will be chosen randomly between the two videos. The entire session will be measured as lasting after approximately 20 min (including explaining the research protocol, putting on, and taking off the SWS).

#### Phase two – Classify the variations of the physiological parameters in people with autism spectrum disorder

**FIGURE 2:**
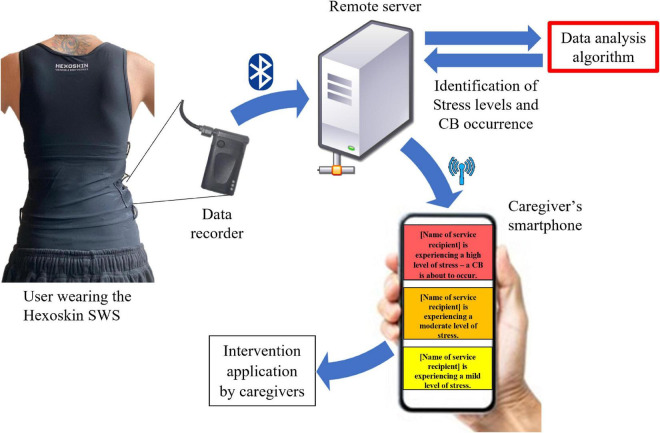
Brief system architecture description. Physiological signals are captured by the Hexoskin smart wearable shirt (SWS) and recorded by the provided data recorder. Recorded data are transferred in real time via Bluetooth technology to a remote server where they are analyzed. If the *ad hoc* developed algorithm detects the incoming data suggesting the occurrence of challenging behavior (CB), the remote server immediately sends a notification alert to the caregiver’s smartphone.

Each participant enrolled in phase two will be asked to wear the Hexoskin SWS for seven consecutive days during waking hours while performing his or her usual daily activities. During the same 7 days, care providers will be asked to report the participants’ status in the behavioral diary. Each evening the data collected by the Hexoskin SWS will be uploaded to an online cloud that is provided with the behavioral diary records of the day. Once the data from all the 10 participants have been collected, a deep learning long short-term memory algorithm (LSTM), which is perceived as a neural network algorithm composed of many layers that the neural network accumulates over time, will be developed in order to understand the variations in the individual’ physiological parameters that occur before a CB and predict the eruption of future CBs. The SWS data will be sent in real-time via Bluetooth technology to a remote server, where it will be classified and analyzed through the developed algorithm, thus yielding a CB behavior alert. In case of “normal” behavior, the algorithm will not classify the existing behavior of the participant as CB. Based on the algorithm mentioned above, a smartphone application will be developed to receive the data. If the algorithm detects the possibility of an incoming CB, a notification will be sent to the care provider’s smartphone to inform on the possible oncoming CB, thereby enabling the implementation of the selected intervention strategy. The system architecture is explained in Figure 2.

#### Phase three – System proof of concept

The developed system prototype and its efficacy will be tested on one participant with ASD for 7 days at the participant’s residence. The participant will wear the Hexoskin SWS during waking hours. Before the beginning of the POC phase, the teachers and caregivers who will interact with the system will be trained on its use, and the authors will be available to clarify any doubts and provide technical assistance during the POC week. At the end of those 7 days, the QUEST 2.0 will be administered to each professional interacting with the system. In addition, a focus group will be carried out with the same care providers, addressing the research questions mentioned above. The focus group will discuss on the information obtained from the QUEST 2.0 administration. In the last part of the focus group, a summation of solutions to each research question will be proposed to the group, and the number of participants who agree or disagree with the proposed summation solutions will be collected.

### Data analyses

#### Section 1

Data collected by the Hexoskin SWS from participants in the OG and CG will be analyzed and compared. From electrocardiogram data, HR will be calculated between two consecutive QRS complexes. Considering the time interval between two QRS complexes as “t,” the corresponding temporal HR will be 60/t (Becker, 2006). In order to remove unwanted artifacts from the HR, a percentage threshold value will be set using the sliding window method, and a minimum allowed peak width will be identified. The removal process will be performed for positive and negative peaks in two rounds. A window will be slid over the HR signal, and its median value will be calculated. The maximum (positive and negative) allowed peak amplitude will be determined for every window by multiplying the window’s median value by a threshold value. The threshold value for positive peaks was set at 30% (for the first removal round) and 25% (for the second removal round) of the window’s mean value. For negative peaks, the threshold value was set at 50% (for the first removal round) and 30% (for the second removal round) of the window’s mean value. Then, all peaks with amplitude larger than the allowed value will be identified from every window. If one of these peaks is found as narrower than the minimum allowed peak width, it will be replaced with the reference window median value. Otherwise, if an identified peak width is larger than the pre-set peak width, its value will be replaced with the maximal allowed HR (for positive peaks) or the minimal allowed HR (for negative peaks). The maximal allowed HR will be calculated with the following formula:"209−(0.7×(Participantage))" (Tanaka et al., 2001). The minimal allowed HR will be 60 beats per minute (Sidhu and Marine, 2020). After removing abnormal peaks, the signal will be filtered with a Gaussian filter with a sigma equal to one. After the HR signal filtering process, the obtained cleaned HR signal will be used to classify the participant’s CBs (herein “stress”) within the following levels: “no stress,” “mild stress,” “moderate stress,” and “high stress.” Each stress level will refer to an HR signal positioned within a specific range of values. The “no stress” level will include the HR values below 90% of the cleaned HR signal’s lowest peak. The “high stress” level will comprise values above 90% of the cleaned HR signal’s highest peak. If this value exceed the maximal allowed HR, it will be substituted with 90% of the maximal allowed HR value. The range left between these two thresholds will be divided into two equal parts (lower and upper half). The HR data positioned in the lower half of this range will be classified as “mild stress” and those positioned in the upper half as “moderate stress.” Each HR value will be classified and assigned with a numerical value corresponding to a stress level (“no stress” = 0, “mild stress” = 1, “moderate stress” = 2, and “high stress” = 3). After acquiring the sequences of the stress levels of all participants of section two, the sequences of the subjects in the OG and CG will be compared using a version of the Smith-Waterman algorithm adapted for the analysis of the obtained data.

#### Section 2

The data gathered by the Hexoskin SWS from participants enrolled in section two will be analyzed as described above. A deep learning algorithm will be developed to predict the incoming participants’ stress levels. To find CB patterns among subjects, the authors intend to construct a classifier based on supervised learning to find anomalies in the subject’s data that might indicate an upcoming CB. Therefore, an LSTM algorithm along with other ML strategies (e.g., RF algorithm) will be guided through pre-defined rule sets to recognize data patterns corresponding to CB occurrence using the data collected by the participants’ caregivers via the behavioral diary and information collected by the SWS.

Long short-term memory algorithm is an extension of the recurrent neural network (RNN). In contrast to the application of machine learning and deep learning, in the process of analyzing and predicting time series information, each data point is based on previous information, which must be examined as well. RNN is the most used network for time series applications since it can form the target vector observing the current input data history, using shared weights among the hiding units of the network across each time step of the data. The authors chose the usage of LSTM, and not RNN, since RNN has one significant problem (the vanishing gradient), where the gradient of the output error is based on previous inputs vanishes when time lags between inputs and errors increases. To overcome this problem, the LSTM is introduced. LSTM is designed as having a memory, which comes into practice by replacing the nonlinear units of RNN in the hidden layers with memory blocks. The network propagates errors throughout the entire network, and as a result, it can learn long-term dependencies and forget unnecessary information based on the data at hand (El Boujnouni and Tali, 2019).

The best classification algorithm will be selected based on the obtained prediction accuracy. The accuracy of the prediction model will be calculated according to common estimation methods such as the confusion matrix, and the area under the curve (AUC) values corresponding to the receiver operator characteristic curve (Ozdenizci et al., 2018; Goodwin et al., 2019; Nuske et al., 2019). These values range from 0.5 to 1 and will be designated as follow: 0.90–1 = excellent, 0.80–0.90 = good, 0.70–0.80 = fair, 0.60–0.70 = poor, and 0.50–0.60 = fail.

#### Section 3

In section three, the themes that will emerge from the focus group will be extracted from the discussion transcription. An axial coding strategy will be applied to calculate the extensiveness of each theme. This qualitative data analysis consists of assigning a reference number to each theme and marking any sentence related to that theme with that number. A reliability check for the code-to-sentence matches will be applied by giving the list of codes to an independent researcher experienced in qualitative analysis and asking him or her to identify the sentence that matches each code (Breen, 2006). The level of agreement with each summation answer to the research questions will be obtained by calculating the percentage of participants that agree with the proposed statement. The authors will discuss the developed answers to the research questions in light of the relevant themes that will have emerged, along with the level of agreement of the discussion group. The participants’ responses to the focus group will be used to improve the usage of the SWS within the context of CB and ASD, as well as further develop the mobile application.

## Results

The expected results for each part of the current investigation are summarized below.

### Phase one

From the data gathered in the study phase one, the authors expect to recognize a higher stress level within the sequences obtained from participants in the OG compared to those from the CG. Although the literature reported a similar HR variation in adults with and without ASD exposed to stressful situations (Bishop-Fitzpatrick et al., 2017; Dijkhuis et al., 2019), adults with ASD are overall experiencing higher stress levels than typically developed peers when exposed to stressors (Gillott and Standen, 2007; Hirvikoski and Blomqvist, 2015; Bishop-Fitzpatrick et al., 2017). Therefore, the authors expect to be able to detect a difference within the stress sequences obtained from the proposed HR classification system during the disturbing video watching using the adapted Smith-Waterman algorithm. On the other hand, the authors expect no difference between the stress sequences obtained from individuals in the OG and CG when the participants watched at the relaxing video.

### Phase two

Although, to the authors’ knowledge, there is no literature reporting the capability of HR analysis in predicting the occurrence of CB in adults with ASD, the reports related to children and youth with ASD are encouraging. Relying on previous findings, the authors expect that the developed LSTM algorithm will be able to predict CBs that are about to occur at least in the upcoming 1 min with an AUC value at least above 0.70 (representing a fair prediction sensitivity) by analyzing the data gathered in the previous 60 s (Ozdenizci et al., 2018; Goodwin et al., 2019; Nuske et al., 2019).

### Phase three

#### Quest 2.0

At the end of the protocol’s POC phase, the QUEST 2.0 questionnaire will be administered to each teacher and caregiver who use the system with the participant with ASD. Within the area related to user satisfaction with the assistive technologies, the authors expect to achieve a high satisfaction value (mean score above 4.0) as the Hexoskin SWS only has to be worn by the participant, and the caregiver side of the system will be integrated into his smartphone (smartphone application). Once the SWS and the smartphone are paired with the remote server, no other actions are required from the caregiver. Therefore, no safety problems are anticipated, and easy use of the system is expected. Moreover, the smartphone application will provide visual and auditory stimuli related to the participant’s stress level leading to comfort use and the possibility of intervening when a high-stress level is identified.

A high satisfaction score is anticipated concerning the area of provided service (mean score above 4.0) as there will be a training period for all who will interact with the system and the availability to provide technical assistance during the POC week. Moreover, the focus group that will be conducted represents a reasonable opportunity to verify the system’s functioning.

#### Focus group

As to the authors’ knowledge, the current protocol represents the first attempt to conduct a focus group evaluating the experience of caregivers with the use of a smart wearable device to predict the CBs of adults with ASD. It is challenging to rely on previous reports that mainly focus on design suggestions for wearable devices for people with ASD. In the discussion of the first research question (“Did wearing the SWS upset the participants?”), the authors expect that no difficulties will be reported on the selected SWS wearing during the POC week as it is a soft undershirt out of the participant’s direct field of vision. Moreover, garments, such as t-shirts, have been found to be a highly preferred device to be used by individuals with ASD (Koo et al., 2018). However, reflections are anticipated about the individual sensory preferences of each person that may compromise the use of SWS in some people with ASD. Themes similar to this one emerged from a previous focus group related to the design of wearable technologies for people with ASD (Cantin-Garside et al., 2021). Furthermore, concerns can arise related to the hottest times of the year, when wearing a tank top under the shirt may be inappropriate.

Concerning the second, third, and fourth research questions (“Was the system able to detect all relevant CBs?”; “Was the system’s operational speed sufficient to allow the in-time application of appropriate prevention strategies?”; “Has the use of the system reduced the amount of CBs?”), the authors expect that the emerging themes will crosscut them. Anticipated themes relate to the different CBs that can occur and the system’s ability to predict all of them. Moreover, considerations are expected about the usefulness of the classification system’s ability to reflect the current participant status and how the real-time knowledge of his arousal level changes the caregivers’ carrying strategies. Finally, anticipated themes comprise the discussion of the usefulness of the time with which the CB is predicted. Reflections may emerge about whether the prediction time is sufficient or not to implement the appropriate CB prevention strategies.

During the fifth research question discussion, one anticipated theme relates to the possibility of using the proposed system in several environments, as the current architecture requires a Bluetooth connection with a server nearby. Moreover, reflections may occur about the benefit of a smaller wearable device, which is less recognizable by the participant, and the possibility of having a wider prediction window. The system’s availability in all the participant’s daily living environments can help the prevention of during the whole day. A smaller device can improve the wearability of the system, increasing the acceptability of the device. Finally, a wider prediction window may be required in some cases for appropriate prevention strategies application.

## Discussion

Many individuals with ASD present aggressive or disruptive CB, negatively affecting the quality of life of the person presenting the CBs. CBs can also reduce the possibility of receiving a proper education, social participation, and job opportunities for the person with ASD. Although numerous interventions have been proposed in the literature regarding how to cope with such behaviors, to date, most of them have not been found to affect CBs in a significant manner positively. Therefore, there is a need for effective strategies to support such interventions that can anticipate oncoming CBs. The results obtained from the analysis described in “Section 1” will deepen the knowledge related to the relationship between HR and stress levels in adults with ASD. Moreover, to the authors’ best knowledge, this study represents the first attempt of using SWSs and physiological parameters to predict CBs of adults with ASD. The results obtained from the prediction algorithm development will lay the foundation for expanding the field of study of CBs prediction through ML techniques to the adult population with ASD. The availability of an effective strategy to anticipate the CBs occurrence will allow the caregivers to intervene, applying the adequate procedure to reduce the person’s stress level and avoid the behavioral meltdown. Such technology can potentially improve the quality of life of people with ASD presenting aggressive and disruptive CBs and their peers, care providers, and healthcare professionals. Moreover, the proposed system will be cost-effective, easy to use even for non-experts, and widely accessible. Furthermore, results that will be obtained at the end of this project can assist in the further development of wearable devices to predict CB, a field of growing interest among health and medical researchers with the potential to help other populations presenting CBs, such as those with intellectual and developmental disabilities, dementia, and other mental challenges. Finally, the mixed-method design proposed for phase three of the protocol will involve care providers and healthcare professionals who handle CB daily. Their participation will allow for the integration of their clinical experience and perceived needs and will provide valuable information to be considered for developing the current application and future similar devices. The current method presents some limitations. First, one participant only will be included in the POC phase. However, although this choice can limit the external validity of the results obtained in the POC phase, it will provide initial data on the usability of the developed system. Moreover, the videos that were chosen for the study phase one were not previously validated to elicit the desired stress increase (or reduction) in the population enrolled in the study (typically developed adults and adults with ASD).

## Data availability statement

The original contributions presented in this study are included in the article/supplementary material, further inquiries can be directed to the corresponding author.

## Ethics statement

The studies involving human participants were reviewed and approved by the Ariel University Institutional Review Board (AU-HEA-ML-20201203), Asaf Harofe Institutional Review Board (0136-21-ASF), and the Israeli Ministry of Health (MOH_2022-01-25_010570). The patients/participants provided their written informed consent to participate in this study.

## Author contributions

MZ and ML obtained the funds for the current research project. MZ, RT, and ML designed the protocol and selected the SWS to be used. MZ, RT, and HH identified the adequate data analysis for the algorithm development and wrote the software requirements specification for the application interfaces. MZ and HH defined the design requirements for the application implementation phase. ML and AR defined the methodology for the clinical part of the data collection (behavioral diary and focus group) and were involved in the data collection section of the proposed research. AR and HH wrote the protocol text. All authors read the protocol draft and suggested improvement until consensus was reached.

## Footnotes

1
https://www.hexoskin.com

## References

[B1] American Psychiatric Association (2013). *Diagnostic and Statistical Manual of Mental Disorders.* 5th Edn. Washington, DC: American Psychiatric Association, 10.1176/appi.books.9780890425596

[B2] Arnold S. Reed P. (2016). Reading assessments for students with ASD: a survey of summative reading assessments used in special educational schools in the UK. *Br. J. Special Educ.* 43 122–141. 10.1111/1467-8578.12127

[B3] Baker A. E. Z. Lane A. Angley M. T. Young R. L. (2008). The relationship between sensory processing patterns and behavioural responsiveness in autistic disorder: A pilot study. *J. Autism. Dev. Disord.* 38 867–875. 17899349 10.1007/s10803-007-0459-0

[B4] Banaee H. Ahmed M. U. Loutfi A. (2013). Data mining for wearable sensors in health monitoring systems: A review of recent trends and challenges. *Sensors* 13 17472–17500. 10.3390/s131217472 24351646 PMC3892855

[B5] Baron-Cohen S. Scott F. J. Allison C. Williams J. Bolton P. Matthews F. E. (2009). Prevalence of autism-spectrum conditions: UK school-based population study. *Br. J. Psychiatry* 194 500–509. 10.1192/BJP.BP.108.059345 19478287

[B6] Barton E. E. Reichow B. Schnitz A. Smith I. C. Sherlock D. (2015). A systematic review of sensory-based treatments for children with disabilities. *Res. Dev. Disabil.* 37 64–80. 10.1016/J.RIDD.2014.11.006 25460221

[B7] Becker D. E. (2006). Fundamentals of electrocardiography interpretation. *Anesth. Prog.* 53 53–64. 10.2344/0003-3006200653[53:FOEI]2.0.CO;2 16863387 PMC1614214

[B8] Berg W. K. Peck S. Wacker D. P. Harding J. McComas J. Richman D. (2000). The effects of presession exposure to attention on the results of assessments of attention as a reinforcer. *J. Appl. Behav. Anal.* 33 463–477. 10.1901/jaba.2000.33-463 11214023 PMC1284271

[B9] Bishop-Fitzpatrick L. Minshew N. J. Eack S. M. (2014). “A systematic review of psychosocial interventions for adults with autism spectrum disorders,” in *Adolescents and Adults with Autism Spectrum Disorders*, Volkmar F. R. Reichow B. McPartland J. C. (Berlin: Springer), 315–327. 10.1007/978-1-4939-0506-5_16 PMC3508309 22825929

[B10] Bishop-Fitzpatrick L. Minshew N. J. Mazefsky C. A. Eack S. M. (2017). Perception of life as stressful, not biological response to stress, is associated with greater social disability in adults with autism spectrum disorder. *J. Autism Dev. Disord.* 47 1–16. 10.1007/s10803-016-2910-6 27696184 PMC5225258

[B11] Blacher J. McIntyre L. L. (2006). Syndrome specificity and behavioural disorders in young adults with intellectual disability: cultural differences in family impact. *J. Intel. Disabil. Res.* 50 184–198. 10.1111/J.1365-2788.2005.00768.X 16430730

[B12] Black M. H. Milbourn B. Chen N. T. M. McGarry S. Wali F. Ho A. S. V. (2020). The use of wearable technology to measure and support abilities, disabilities and functional skills in autistic youth: a scoping review. *Scand. J. Child Adolesc. Psychiatr. Psychol.* 8 48–69. 10.21307/sjcapp-2020-006 33520778 PMC7685500

[B13] Blankenship K. Erickson C. A. McDougle C. J. (2010). Pharmacotherapy of Autism and related disorders. *Psychiatr. Ann.* 40 203–209. 10.3928/00485713-20100330-06

[B14] Breen R. L. (2006). A practical guide to focus-group research. *J. Geogr. High. Educ.* 30 463–475. 10.1080/03098260600927575

[B15] Bremer E. Crozier M. Lloyd M. (2016). A systematic review of the behavioural outcomes following exercise interventions for children and youth with autism spectrum disorder. *Autism* 20 899–915. 10.1177/1362361315616002 26823546

[B16] Cantin-Garside K. D. Nussbaum M. A. White S. W. Kim S. Kim C. do Fortes D. M. G. (2021). Understanding the experiences of self-injurious behavior in autism spectrum disorder: Implications for monitoring technology design. *J. Am. Med. Inform. Assoc.* 28 303–310. 10.1093/jamia/ocaa169 32974678 PMC7883971

[B17] Case-Smith J. Weaver L. L. Fristad M. A. (2015). A systematic review of sensory processing interventions for children with autism spectrum disorders. *Autism* 19 133–148. 10.1177/1362361313517762 24477447

[B18] Chiang H. M. (2008). Expressive communication of children with autism: the use of challenging behaviour. *J. Intel. Disabil. Res.* 52 966–972. 10.1111/J.1365-2788.2008.01042.X 18205752

[B19] Cohen I. L. Yoo J. H. Goodwin M. S. Moskowitz L. (2011). “Assessing challenging behaviors in autism spectrum disorders: prevalence, rating scales, and autonomic indicators,” in *International Handbook of Autism and Pervasive Developmental Disorders*, eds Matson J. Sturmey P. (New York, NY: Springer New York), 247–270. 10.1007/978-1-4419-8065-6_15

[B20] Cooper J. O. Heron T. E. Heward W. L. (2020). *Applied Behavior Analysis.* 3rd Edn. Harlow: Pearson.

[B21] Cotelli M. Manenti R. Zanetti O. (2012). Reminiscence therapy in dementia: A review. *Maturitas* 72 203–205. 10.1016/j.maturitas.2012.04.008 22607813

[B22] Demers L. Weiss-Lambrou R. Ska B. (2002). The Quebec User Evaluation of Satisfaction with Assistive Technology (QUEST 2.0): An overview and recent progress. *Technol. Disabil.* 14 101–105. 10.3233/TAD-2002-14304

[B23] Demers L. Weiss-Lambrou R. Ska B. Demers L. (2000). Item analysis of the quebec user evaluation of satisfaction with assistive technology (QUEST). *Assist. Technol.* 12 96–105. 10.1080/10400435.2000.10132015 11508406

[B24] Didden R. Korzilius H. Van Oorsouw W. Sturmey P. (2006). Behavioral treatment of challenging behaviors in individuals with mild mental retardation: Meta-analysis of single-subject research. *Am. J. Mental Retard.* 111 290–298. 10.1352/0895-80172006111[290:BTOCBI]2.0.CO;2 16792430

[B25] Dijkhuis R. R. Ziermans T. van Rijn S. Staal W. Swaab H. (2019). Emotional arousal during social stress in young adults with autism: insights from heart rate, heart rate variability and self-report. *J. Autism Dev. Disord.* 49 2524–2535. 10.1007/s10803-019-04000-5 30945093 PMC6546666

[B26] Doyle K. L. Morgan E. E. Morris S. Smith D. M. Little S. Iudicello J. E. (2013). Real-world impact of neurocognitive deficits in acute and early HIV infection. *J. Neurovirol.* 19 565–573. 10.1007/s13365-013-0218-2 24277439 PMC3865175

[B27] Eggermont L. H. P. Scherder E. J. A. (2006). Physical activity and behaviour in dementia: A review of the literature and implications for psychosocial intervention in primary care. *Dementia* 5 411–428. 10.1177/1471301206067115

[B28] El Boujnouni I. Tali A. (2019). Heart rate variability prediction based on the combination of wavelet decomposition and LSTM networks. *Int. J. Sci. Eng. Res.* 10 61–65.

[B29] Emerson E. (2001). *Challenging Behaviour: Analysis and Intervention in People with Severe Intellectual Disabilities.* 2nd Edn. Cambridge: Cambridge University Press.

[B30] Erturk B. Machalicek W. Drew C. (2018). Self-injurious behavior in children with developmental disabilities: a systematic review of behavioral intervention literature. *Behav. Modif.* 42 498–542. 10.1177/0145445517741474 29179569

[B31] Fakhoury N. Wilhelm N. Sobota K. F. Kroustos K. R. (2017). Impact of music therapy on dementia behaviors: A literature review. *Consult. Pharm.* 32 623–628. 10.4140/TCP.N.2017.623 28992823

[B32] Felce D. Perry J. Lowe K. Jones E. (2011). The impact of autism or severe challenging behaviour on lifestyle outcome in community housing. *J. Appl. Res. Intel. Disabil.* 24 95–104. 10.1111/J.1468-3148.2010.00571.X

[B33] Ferguson J. Craig E. A. Dounavi K. (2019). Telehealth as a model for providing behaviour analytic interventions to individuals with autism spectrum disorder: a systematic review. *J. Autism. Dev. Disord.* 49 582–616. 10.1007/S10803-018-3724-5/FIGURES/5 30155578 PMC6373531

[B34] Fettig A. Barton E. E. (2014). Parent implementation of function-based intervention to reduce children’s challenging behavior: a literature review. *Topics Early Child. Spec. Educ.* 34 49–61. 10.1177/0271121413513037

[B35] Flores J. G. Alonso C. G. (1995). Using focus groups in educational research: exploring teachers’ perspectives on educational change. *Eval. Rev.* 19 84–101. 10.1177/0193841X9501900104

[B36] Forbes D. Forbes S. C. Blake C. M. Thiessen E. J. Forbes S. (2015). Exercise programs for people with dementia. *Cochrane Database Syst. Rev.* 2015 195–196. 10.1002/14651858.CD006489.pub4 25874613 PMC9426996

[B37] Francese R. Yang X. (2021). Supporting autism spectrum disorder screening and intervention with machine learning and wearables: a systematic literature review. *Complex Intel. Syst.* 8 3659–3674. 10.1007/S40747-021-00447-1

[B38] Gerow S. Hagan-Burke S. Rispoli M. Gregori E. Mason R. Ninci J. (2018). A Systematic Review of Parent-Implemented Functional Communication Training for Children With ASD. *Behav. Modif.* 42 335–363. 10.1177/0145445517740872 29199433

[B39] Gillott A. Standen P. J. (2007). Levels of anxiety and sources of stress in adults with autism. *J. Intel. Disabil.* 11 359–370. 10.1177/1744629507083585 18029412

[B40] Gold C. Wigram T. Elefant C. (2006). Music therapy for autistic spectrum disorder. *Cochrane Database Syst. Rev.* 2006:CD004381. 10.1002/14651858.CD004381.PUB2/INFORMATION/EN 16625601

[B41] Goldstein H. (2002). Communication intervention for children with autism: a review of treatment efficacy. *J. Autism Dev. Disord.* 32 373–396. 10.1023/A:1020589821992 12463516

[B42] Goodwin M. S. Mazefsky C. A. Ioannidis S. Erdogmus D. Siegel M. (2019). Predicting aggression to others in youth with autism using a wearable biosensor. *Autism Res.* 12 1286–1296. 10.1002/aur.2151 31225952 PMC6988899

[B43] Goodwin M. S. Özdenizci O. Cumpanasoiu C. Tian P. Guo Y. Stedman A. (2018). “Predicting imminent aggression onset in minimally-verbal youth with autism spectrum disorder using preceding physiological signals,” in *Proceedings of the 12th EAI International Conference on Pervasive Computing Technologies for Healthcare* (New York, NY: ACM), 201–207. 10.1145/3240925.3240980 PMC6230252 30420938

[B44] Gregori E. Wendt O. Gerow S. Peltier C. Genc-Tosun D. Lory C. (2020). Functional communication training for adults with autism spectrum disorder: a systematic review and quality appraisal. *J. Behav. Educ.* 29 42–63. 10.1007/s10864-019-09339-4

[B45] Gulshan V. Peng L. Coram M. Stumpe M. C. Wu D. Narayanaswamy A. (2016). Development and validation of a deep learning algorithm for detection of diabetic retinopathy in retinal fundus photographs. *JAMA* 316 2402–2410. 10.1001/jama.2016.17216 27898976

[B46] Hirvikoski T. Blomqvist M. (2015). High self-perceived stress and poor coping in intellectually able adults with autism spectrum disorder. *Autism* 19 752–757. 10.1177/1362361314543530 25073750

[B47] Holden B. Gitlesen J. P. (2006). A total population study of challenging behaviour in the county of Hedmark, Norway: Prevalence, and risk markers. *Res. Dev. Disabil.* 27 456–465. 10.1016/J.RIDD.2005.06.001 16137857

[B48] Hutt C. Hutt S. J. (1965). Effects of environmental complexity on stereotyped behaviours of children. *Anim. Behav.* 13 1–4. 10.1016/0003-3472(65)90064-3

[B49] Imbiriba T. Cumpanasoiu D. C. Heathers J. Ioannidis S. Erdomuş, D., and Goodwin M. S. (2020). Biosensor prediction of aggression in youth with autism using kernel-based methods. *ACM Int. Conf. Proceeding Ser.* 88–93. 10.1145/3389189.3389199

[B50] Inoue M. (2019). Assessments and interventions to address challenging behavior in individuals with intellectual disability and autism spectrum disorder in Japan: a consolidated review. *Yonago Acta Med.* 62 169–181. 10.33160/YAM.2019.06.001 31320821 PMC6584262

[B51] James R. Sigafoos J. Green V. A. Lancioni G. E. O’Reilly M. F. Lang R. (2015). Music therapy for individuals with autism spectrum disorder: a systematic review. *Rev. J. Autism Dev. Disord.* 2 39–54. 10.1007/s40489-014-0035-4

[B52] Jang J. Dixon D. R. Tarbox J. Granpeesheh D. (2011). Symptom severity and challenging behavior in children with ASD. *Res. Autism Spectr. Disord.* 5 1028–1032. 10.1016/j.rasd.2010.11.008

[B53] Kinsbourne M. (1980). Do repetitive movement patterns in children and animals serve a dearousing function? *J. Dev. Behav. Pediatr.* 1 39–42. 10.1097/00004703-198003000-00009 6941968

[B54] Kitzinger J. (1994). The methodology of Focus Groups: the importance of interaction between research participants. *Sociol. Health Illn.* 16 103–121. 10.1111/1467-9566.ep11347023

[B55] Klusek J. Roberts J. E. Losh M. (2015). Cardiac autonomic regulation in autism and Fragile X syndrome: A review. *Psychol. Bull.* 141 141–175. 10.1037/a0038237 25420222 PMC4293203

[B56] Knapp M. Romeo R. Beecham J. (2009). Economic cost of autism in the UK. *Autism* 13 317–336. 10.1177/1362361309104246 19369391

[B57] Koo S. H. Gaul K. Rivera S. Pan T. Fong D. (2018). Wearable technology design for autism spectrum disorders. *Arch. Design Res.* 31 37–55. 10.15187/adr.2018.02.31.1.37

[B58] Lang R. O’Reilly M. Healy O. Rispoli M. Lydon H. Streusand W. (2012). Sensory integration therapy for autism spectrum disorders: A systematic review. *Res. Autism Spectr. Disord.* 6 1004–1018. 10.1016/j.rasd.2012.01.006

[B59] Leong H. M. Carter M. Stephenson J. (2015). Systematic review of sensory integration therapy for individuals with disabilities: Single case design studies. *Res. Dev. Disabil.* 47 334–351. 10.1016/J.RIDD.2015.09.022 26476485

[B60] Lequia J. MacHalicek W. Rispoli M. J. (2012). Effects of activity schedules on challenging behavior exhibited in children with autism spectrum disorders: A systematic review. *Res. Autism Spectr. Disord.* 6 480–492. 10.1016/j.rasd.2011.07.008

[B61] Levine T. P. Conradt E. Goodwin M. S. Sheinkopf S. J. Lester B. (2014). “Psychophysiological arousal to social stress in autism spectrum disorders,” in *Comprehensive Guide to Autism*, eds Patel V. B. Preedy V. R. Martin C. R. (New York, NY: Springer New York), 1177–1193. 10.1007/978-1-4614-4788-7_66

[B62] Lim J. M. (2019). Emotion regulation and intervention in adults with autism spectrum disorder: a synthesis of the literature. *Adv. Autism* 6 48–62. 10.1108/AIA-12-2018-0050

[B63] Lindgren S. Wacker D. Suess A. Schieltz K. Pelzel K. Kopelman T. (2016). Telehealth and autism: treating challenging behavior at lower cost. *Pediatrics* 137 S167–S175. 10.1542/PEDS.2015-2851O 26908472 PMC4727312

[B64] Lloyd B. P. Kennedy C. H. (2014). Assessment and treatment of challenging behaviour for individuals with intellectual disability: a research review. *J. Appl. Res. Intel. Disabil.* 27 187–199. 10.1111/JAR.12089 24464965

[B65] Lydon S. Healy O. O’Reilly M. McCoy A. (2013). A systematic review and evaluation of response redirection as a treatment for challenging behavior in individuals with developmental disabilities. *Res. Dev. Disabil.* 34 3148–3158. 10.1016/J.RIDD.2013.06.010 23886757

[B66] Lydon S. Healy O. Reed P. Mulhern T. Hughes B. M. Goodwin M. S. (2016). A systematic review of physiological reactivity to stimuli in autism. *Dev. Neurorehabil.* 19 335–355. 10.3109/17518423.2014.971975 25356589

[B67] Machalicek W. O’Reilly M. F. Beretvas N. Sigafoos J. Lancioni G. E. (2007). A review of interventions to reduce challenging behavior in school settings for students with autism spectrum disorders. *Res. Autism Spectr. Disord.* 1 229–246. 10.1016/J.RASD.2006.10.005

[B68] Machalicek W. Raulston T. Knowles C. Ruppert T. Carnett A. Alresheed F. (2016). “Challenging behavior,” in *Comorbid Conditions Among Children with Autism Spectrum Disorders*, Ed. Matson J. L. (Cham: Springer), 137–170. 10.1007/978-3-319-19183-6_6

[B69] MacNaul H. L. Neely L. C. (2018). Systematic review of differential reinforcement of alternative behavior without extinction for individuals with autism. *Behav. Modif.* 42 398–421. 10.1177/0145445517740321 29117712

[B70] Malone R. P. Gratz S. S. Delaney M. A. Hyman S. B. (2005). Advances in drug treatments for children and adolescents with autism and other pervasive developmental disorders. *CNS Drugs* 19 923–934. 10.2165/00023210-200519110-00003/FIGURES/TAB1 16268664

[B71] Masino A. J. Forsyth D. Nuske H. Herrington J. Pennington J. Kushleyeva Y. (2019). “M-Health and autism: Recognizing stress and anxiety with machine learning and wearables data,” in *Proceedings of the IEEE Symposium on Computer-Based Medical Systems 2019-June*, Shenzhen, 714–719. 10.1109/CBMS.2019.00144

[B72] Matson J. L. Wilkins J. Macken J. (2008). The relationship of challenging behaviors to severity and symptoms of autism spectrum disorders. *J. Ment. Health Res. Intellect. Disabil.* 2 29–44. 10.1080/19315860802611415

[B73] McDonnell A. Sturmey P. Oliver C. Cunningham J. Hayes S. Galvin M. (2008). The effects of staff training on staff confidence and challenging behavior in services for people with autism spectrum disorders. *Res. Autism. Spectr. Disord.* 2 311–319. 10.1016/J.RASD.2007.08.001

[B74] McPheeters M. L. Warren Z. Sathe N. Bruzek J. L. Krishnaswami S. Jerome R. N. (2011). A systematic review of medical treatments for children with autism spectrum disorders. *Pediatrics* 127 e1312–e1321. 10.1542/peds.2011-0427 21464191

[B75] Mirenda P. (1997). Supporting individuals with challenging behavior through functional communication training and AAC: research review. *Augment. Alter. Commun.* 13 207–225. 10.1080/07434619712331278048

[B76] Mukaddes N. M. Topcu Z. (2006). Case report: homicide by a 10-year-old girl with autistic disorder. *J. Autism Dev. Disord.* 36 471–474. 10.1007/S10803-006-0087-0 16604294

[B77] Murphy G. H. Beadle-Brown J. Wing L. Gould J. Shah A. Holmes N. (2005). Chronicity of challenging behaviours in people with severe intellectual disabilities and/or autism: a total population sample. *J. Autism Dev. Disord.* 35 405–418. 10.1007/S10803-005-5030-2 16134027

[B78] Murphy K. P. (2012). *Machine Learning: A Probabilistic Perspective.* Cambridge, MA: MIT press.

[B79] Murphy O. Healy O. Leader G. (2009). Risk factors for challenging behaviors among 157 children with autism spectrum disorder in Ireland. *Res. Autism. Spectr. Disord.* 3 474–482. 10.1016/j.rasd.2008.09.008

[B80] Neal M. Barton Wright P. (2003). Validation therapy for dementia. *Cochrane Database Syst. Rev.* 2003:CD001394 10.1002/14651858.CD001394 12917907

[B81] Nissen J. M. J. F. Haveman M. J. (1997). Mortality and avoidable death in people with severe self-injurious behaviour: results of a Dutch study. *J. Intel. Disabil. Res.* 41 252–257. 10.1046/J.1365-2788.1997.04545.X 9219074

[B82] Nuske H. J. Finkel E. Hedley D. Parma V. Tomczuk L. Pellecchia M. (2019). Heart rate increase predicts challenging behavior episodes in preschoolers with autism. *Stress* 22 303–311. 10.1080/10253890.2019.1572744 30822219

[B83] O’Donnell S. Deitz J. Kartin D. Nalty T. Dawson G. (2012). Sensory processing, problem behavior, adaptive behavior, and cognition in preschool children with autism spectrum disorders. *Am. J. Occupat. Ther.* 66 586–594. 10.5014/ajot.2012.004168 22917125

[B84] O’Neil M. E. Freeman M. Christensen V. Telerant R. Addleman A. Kansagara D. (2011). *A Systematic Evidence Review of Non-Pharmacological Interventions for Behavioral Symptoms of Dementia.* Washington, DC: Department of Veterans Affairs. 21634073

[B85] Ogg-Groenendaal M. Hermans H. Claessens B. (2014). A systematic review on the effect of exercise interventions on challenging behavior for people with intellectual disabilities. *Res. Dev. Disabil.* 35 1507–1517. 10.1016/J.RIDD.2014.04.003 24763376

[B86] Ornitz E. M. Ritvo E. R. (1968). Perceptual inconstancy in early infantile autism: the syndrome of early infant autism and its variants including certain cases of childhood schizophrenia. *Arch. Gen. Psychiatry* 18 76–98. 10.1001/ARCHPSYC.1968.01740010078010 4169269

[B87] Ozdenizci O. Cumpanasoiu C. Mazefsky C. Siegel M. Erdogmus D. Ioannidis S. (2018). “Time-series prediction of proximal aggression onset in minimally-verbal youth with autism spectrum disorder using physiological biosignals,” in *Proceedings of the Annual International Conference of the IEEE Engineering in Medicine and Biology Society (IEEE)*, Sydney, 5745–5748. 10.1109/EMBC.2018.8513524 30441641

[B88] Prizant B. M. Wetherby A. M. (2005). “Critical issues in enhancing communication abilities for persons with autism spectrum disorders,” in *Handbook of Autism and Pervasive Developmental Disorders: Assessment, Interventions, and Policy*, eds Volkmar F. R. Paul R. Klin A. Cohen D. (Hoboken, NJ: John Wiley & Sons, Inc.), 925–945. 10.1002/9780470939352.ch10

[B89] Reese R. M. Richman D. M. Belmont J. M. Morse P. (2005). Functional characteristics of disruptive behavior in developmentally disabled children with and without autism. *J. Autism Dev. Disord.* 35 419–428. 10.1007/S10803-005-5032-0 16134028

[B90] Reichow B. Servili C. Yasamy M. T. Barbui C. Saxena S. (2013). Non-specialist psychosocial interventions for children and adolescents with intellectual disability or lower-functioning autism spectrum disorders: a systematic review. *PLoS Med.* 10:e1001572. 10.1371/JOURNAL.PMED.1001572 24358029 PMC3866092

[B91] Rose D. Horne S. Rose J. L. Hastings R. P. (2004). Negative emotional reactions to challenging behaviour and staff burnout: Two replication studies. *J. Appl. Res. Intel. Disabil.* 17 219–223. 10.1111/j.1468-3148.2004.00194.x

[B92] Sawyer A. Lake J. K. Lunsky Y. Liu S. K. Desarkar P. (2014). Psychopharmacological treatment of challenging behaviours in adults with autism and intellectual disabilities: A systematic review. *Res. Autism. Spectr. Disord.* 8 803–813. 10.1016/J.RASD.2014.03.021

[B93] Scherer M. J. (2005). *Living in the State of Stuck: How Technology Impacts the Lives of Persons with Disabilities.* 4th Edn. Brookline, MA: Brookline Books.

[B94] Scotti J. R. Ujcich K. J. Weigle K. L. Holland C. M. Kirk K. S. (1996). Interventions with challenging behavior of persons with developmental disabilities: a review of current research practices. *J. Assoc. Persons Severe Handic.* 21 123–134. 10.1177/154079699602100303

[B95] Seida J. K. Ospina M. B. Karkhaneh M. Hartling L. Smith V. Clark B. (2009). Systematic reviews of psychosocial interventions for autism: An umbrella review. *Dev. Med. Child. Neurol.* 51 95–104. 10.1111/j.1469-8749.2008.03211.x 19191842

[B96] Sidhu S. Marine J. E. (2020). Evaluating and managing bradycardia. *Trends Cardiovasc. Med.* 30 265–272. 10.1016/j.tcm.2019.07.001 31311698

[B97] Simpson K. Keen D. (2011). Music interventions for children with autism: Narrative review of the literature. *J. Autism Dev. Disord.* 41 1507–1514. 10.1007/s10803-010-1172-y 21203898

[B98] Sorensen C. Zarrett N. (2014). Benefits of physical activity for adolescents with autism spectrum disorders: a comprehensive review. *Rev. J. Autism Dev. Disord.* 1 344–353. 10.1007/S40489-014-0027-4/TABLES/4 26744347

[B99] Stephenson J. (2006). Music therapy and the education of students with severe disabilities. *Educ. Train Dev. Disabil.* 41 290–299.

[B100] Subramaniam P. Woods B. (2012). The impact of individual reminiscence therapy for people with dementia: Systematic review. *Expert Rev. Neurother.* 12 545–555. 10.1586/ern.12.35 22550983

[B101] Taj-Eldin M. Ryan C. O’Flynn B. Galvin P. (2018). A review of wearable solutions for physiological and emotional monitoring for use by people with autism spectrum disorder and their caregivers. *Sensors* 18:4271. 10.3390/s18124271 30518133 PMC6308558

[B102] Tanaka H. Monahan K. D. Seals D. R. (2001). Age-predicted maximal heart rate revisited. *J. Am. Coll. Cardiol.* 37 153–156. 10.1016/S0735-1097(00)01054-8 11153730

[B103] Tanner K. Hand B. N. O’Toole G. Lane A. E. (2015). Effectiveness of interventions to improve social participation, play, leisure, and restricted and repetitive behaviors in people with autism spectrum disorder: A systematic review. *Am. J. Occupat. Ther.* 69 6905180010p1–6905180010p12. 10.5014/ajot.2015.017806 26356653

[B104] Vanderkerken L. Heyvaert M. Maes B. Onghena P. (2013). Psychosocial interventions for reducing vocal challenging behavior in persons with autistic disorder: A multilevel meta-analysis of single-case experiments. *Res. Dev. Disabil.* 34 4515–4533. 10.1016/J.RIDD.2013.09.030 24183495

[B105] Wahman C. L. Pustejovsky J. E. Ostrosky M. M. Santos R. M. (2022). Examining the effects of social storiestm on challenging behavior and prosocial skills in young children: a systematic review and meta-analysis. *Topics Early Child Spec. Educ.* 41 267–279. 10.1177/0271121419855692

[B106] Walker V. L. Snell M. E. (2013). Effects of augmentative and alternative communication on challenging behavior: A meta-analysis. *Augment. Alter. Commun.* 29 117–131. 10.3109/07434618.2013.785020 23705814

[B107] Walker V. L. Carpenter M. E. Lyon K. J. Button L. (2021). A meta-analysis of paraprofessional-delivered interventions to address challenging behavior among students with disabilities. *J. Posit Behav. Interv.* 23 68–80. 10.1177/1098300720911147

[B108] Wan Yunus F. Liu K. P. Y. Bissett M. Penkala S. (2015). Sensory-based intervention for children with behavioral problems: a systematic review. *J. Autism Dev. Disord.* 45 3565–3579. 10.1007/S10803-015-2503-9/TABLES/4 26092640

[B109] Watling R. Hauer S. (2015). Effectiveness of Ayres sensory integration^®^ and sensory-based interventions for people with autism spectrum disorder: a systematic review. *Am. J. Occupat. Ther.* 69 6905180030p1–6905180030p12. 10.5014/ajot.2015.018051 26356655

[B110] Weston R. Hodges A. Davis T. N. (2018). Differential reinforcement of other behaviors to treat challenging behaviors among children with autism: a systematic and quality review. *Behav. Modif.* 42 584–609. 10.1177/0145445517743487 29169240

[B111] Woodbury-Smith M. R. Clare I. C. H. Holland A. J. Kearns A. (2006). High functioning autistic spectrum disorders, offending and other law-breaking: Findings from a community sample. *J. Forensic Psychiatry Psychol.* 17 108–120. 10.1080/14789940600589464

[B112] Zetteler J. (2008). Effectiveness of simulated presence therapy for individuals with dementia: A systematic review and meta-analysis. *Aging Ment. Health* 12 779–785. 10.1080/13607860802380631 19023729

[B113] Zheng Z. K. Staubitz J. E. Weitlauf A. S. Staubitz J. Pollack M. Shibley L. (2021). A predictive multimodal framework to alert caregivers of problem behaviors for children with ASD (PreMAC). *Sensors* 21:370. 10.3390/S21020370 33430371 PMC7826816

